# When obstetrics–gynecology specialists need to call an ophthalmologist urgently: a case report

**DOI:** 10.1186/s13256-021-03087-8

**Published:** 2021-10-21

**Authors:** S. Masmejan, Y. Guex-Crosier, C. Diserens, M. Vouga, A. S. Clottu, C. Ribi, P. Mathevet, M. Jacot-Guillarmod

**Affiliations:** 1 Service de Gynécologie et Obstétrique, Département Femme-Mère-Enfant, Centre Hospitalier Universitaire Vaudois, CHUV, 1011 Lausanne, Switzerland; 2 Jules-Gonin Eye Hospital, Ophthalmogy Department, FAA, University of Lausanne, Lausanne, Switzerland; 3 Service d’immunologie et d’allergie, Département de Médecine, Centre Hospitalier Universitaire Vaudois, CHUV, 1011 Lausanne, Switzerland

**Keywords:** Behçet, Genital ulcers, Ophthalmologic emergency, Macular occlusion, Immunosuppressors

## Abstract

**Background:**

We report here a case of a healthy 23-year-old female patient who was assessed at the gynecology emergency department for genital ulcers, fever, and blurred vision. After suspicion of herpes simplex virus-2 lesions, the diagnosis of Behçet’s disease was made. We report this case with the aim of including Behçet’s disease in the differential diagnosis of genital ulcers, and emphasize the emergency of the vision loss that can be irreversible.

**Case presentation:**

A healthy 23-year-old European female patient was assessed by gynecology in the emergency department for genital lesions associated with fever and blurred vision. At first, these lesions were suspected to be primary herpes simplex virus-2 infection One day later, she experienced decreased visual acuity in both eyes. After 4 days of worsening genital ulcers and persistent blurred vision, the patient was referred to the ophthalmology department. Fundoscopic examination showed retinal hemorrhages that were consistent with the first presentation of Behçet’s disease.

**Conclusions:**

This case demonstrates that genital ulcers can be the very initial symptom of this ophthalmologic emergency. The differential diagnosis of genital ulcers is challenging. Behçet’s disease should be included, especially when associated with systemic or ocular manifestations, and should be considered an emergency for the gynecologist to prevent long-term vision loss.

## Introduction

Behçet’s disease is a rare inflammatory disorder, characterized by vascular injuries and autoimmune dysfunction with multisystemic complications. Recurrent inflammatory attacks targeting oral and genital mucous membranes, eyes, and, less frequently but also gastrointestinal tract, central nervous system, and large vessels require multidisciplinary management [1]. A prompt diagnosis is essential to prevent extremely severe complications such as blindness, which affects 20–25% of patients with Behçet’s disease presenting ocular symptoms [1, 2]. To illustrate the issues of multidisciplinary management of Behçet’s disease, we discuss in this report a rare cause of genital ulcerations associated with visual impairment and generalized symptoms in a young female patient for whom the consequences may have been severe if it had not been recognized and treated early. The patient provided her verbal consent for the publication of this case report and images.

## Case description

A healthy 23-year-old Western-European female patient consulted the gynecological emergency service for vulvar pain and edema with small vulvar lesions, associated with febrile episodes up to 39.5 °C. All symptoms appeared the day prior to presentation, starting with vulvar swelling. Family history revealed no similar symptoms, sexual history was unremarkable, and no recent travel was reported. Clinical examination showed diffuse vulvar erythema and edema with several small slightly erosive papules, and one small (< 1 cm) painless inguinal lymph node. Based on the clinical examination and the patient’s symptoms, a diagnosis of primary herpes simplex virus-2 (HSV-2) infection was suspected. Polymerase chain reaction (PCR) swab of the genital ulcerative lesions was performed, and the patient was discharged with antiviral treatment (valaciclovir) and nonsteroidal antiinflammatory pain medications.

One day later, she consulted the general emergency department complaining of blurred vision in both eyes, headache, persistent genital lesions, lumbar pain, and recurrent fever (39 °C). Neurological examination was normal, with the exception of bilateral decreased visual acuity. The presence of retinal hemorrhages was suspected. C-reactive protein (CRP) was 204 mg/L and leukocytes 13.9 G/L. Repetitive hemocultures, white blood cell count, protein PCR and culture of cerebrospinal fluid from a lumbar puncture, PCR samples of vulvar lesions (HSV, chlamydia, and gonococcus), and serology results (Epstein-Barr virus (EBV, syphilis, Human Immunodeficiency Virus HIV)) were all negative. Given the suspicion of retinal lesions, the patient was advised to rapidly consult an ophthalmologist. As blurred vision can be a possible side effect of valaciclovir, the antiviral treatment was switched to acyclovir, and the patient was discharged.

**Fig. 1:**
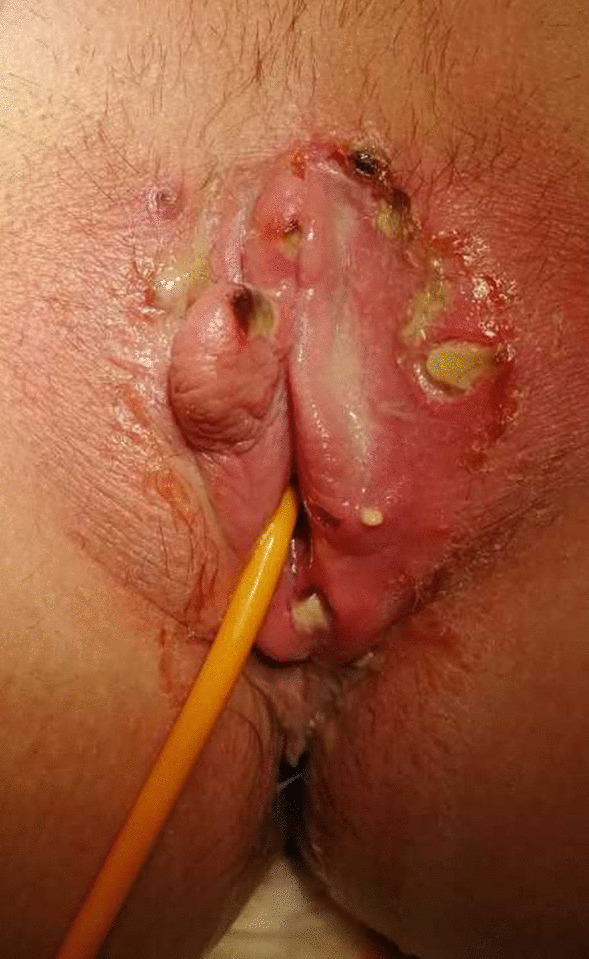
Multiple painful ulcerative vulvar lesions

Four days after initial symptoms, due to ongoing worsening of the genital lesions that had progressed to multiple painful ulcerations (Fig. 1) and were accompanied by dysuria, urinary retention, and persistent blurred vision, the patient consulted the gynecological emergency service. She had not consulted an ophthalmologist in the meantime. The lesions had progressed from papules to deep ulcerations with scabs (Fig. 1). The course of the symptoms is presented in Table 1.

**Table 1:** Timeline of the episodes of care

	Day 1	Day 2	Day 4
Place	Gynecology ER	General ER	Gynecology ER
Clinical findings	Vulvar papules and edema, fever 39.5 °C, inguinal adenopathy	Blurred vision, persistent genital lesions, lumbar pain, recurrent fever 39 °C; decreased visual acuity, suspicion of retinal hemorrhages	Painful deep genital ulcerations, dysuria, urinary retention, persistence of blurred vision; patient is concerned about vulvar lesions; has not been to an ophthalmologist yet Fundoscopic examination shows parafoveolar microhemorrhages and whitish lesions of the retina; OCTA: microangiopathy of the retina
Laboratory results	PCR swabs for HSV of vulvar lesions sent to the laboratory	Leukocytosis 13.9 G/L, CRP 204 mg/L, CSF culture, hemocultures, serologies all normal; PCR swabs of vulvar lesions negative	–
Suspected diagnosis	HSV-2 primo-infection	Possible side effect of valaciclovir	Inaugural Behçet’s disease
Decision	Discharged with oral valaciclovir and NSAID	Switch to acyclovir, patient advised to consult an ophthalmologist	Admitted for in-patient stay, urinary catheter, pulsed methylprednisolone 500 mg/day

On the first line, day after onset of first symptoms

*ER* emergency room, *OCTA* optical coherence tomography angiography, *PCR* polymerase chain reaction, *CSF* cerebrospinal fluid, *HSV* herpes simplex virus, *NSAID* nonsteroidal antiinflammatory drugs, *LC* leucocytes, *CRP* C reactive protein

### Diagnostic assessment, details on the therapeutic intervention, follow-up, and outcomes

The differential diagnosis of the genital ulcerations included HSV, syphilis, chancroid, granuloma inguinale, lymphogranuloma venereum, HIV, Lipschütz ulcer, Crohn disease, and Behçet’s disease.

**Fig. 2:**
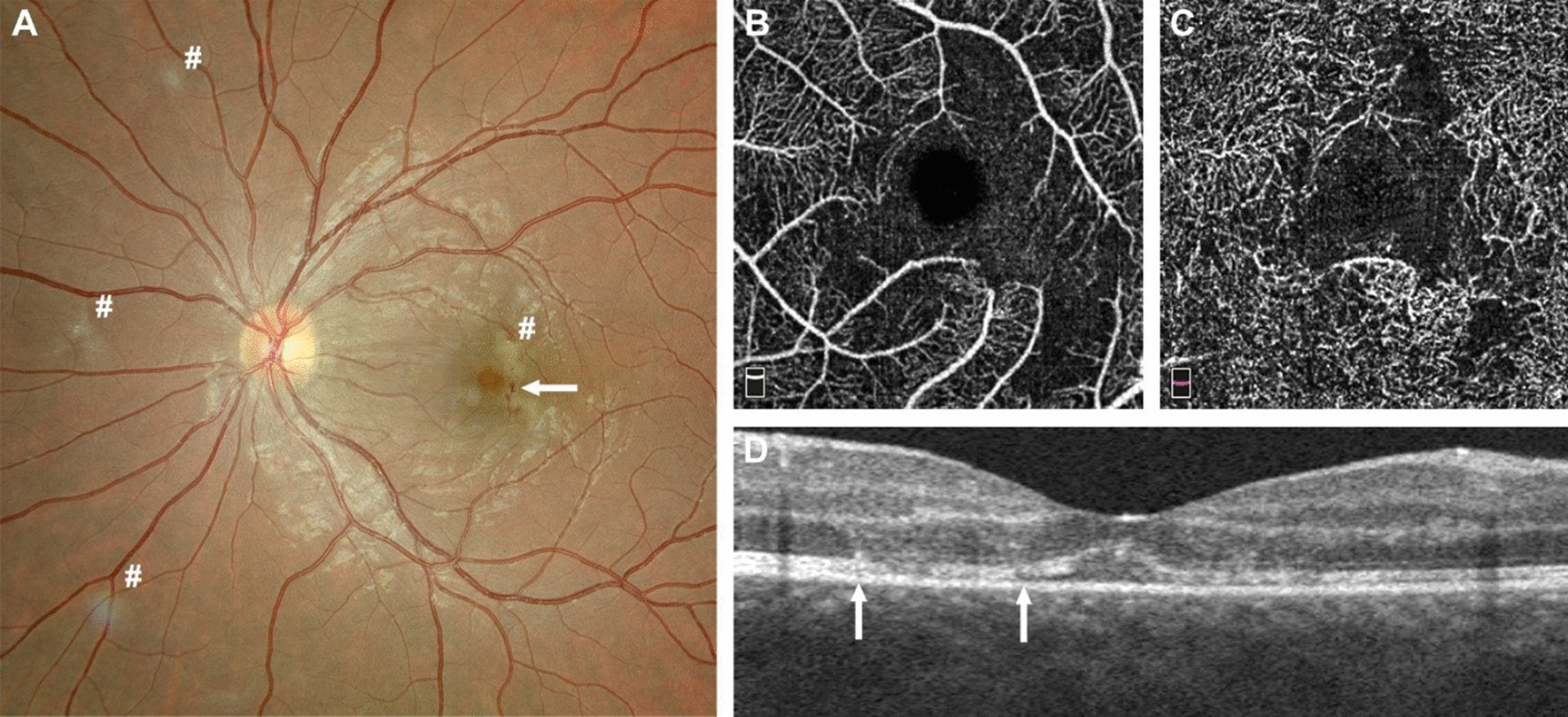
**A** Fundus photography of the left eye: presence of hemorrhages in the posterior pole (arrow), zones of focal ischemia vaso-occlusive retinitis also present nasally  and around the macula (# in the picture). **B**–**D** Optical coherence tomography angiography (OCTA, Optovue imaging) revealing alterations of the superficial plexus of the retina (**B**) and of the deep retinal plexus (**C**); note the disappearance of the vessels due to occlusive vasculitis. **D** Horizontal optical coherence tomography (OCT, Optovue imaging) revealing alterations of the retinal layers (between the arrows) due to local capillary ischemia

**Fig. 3:**
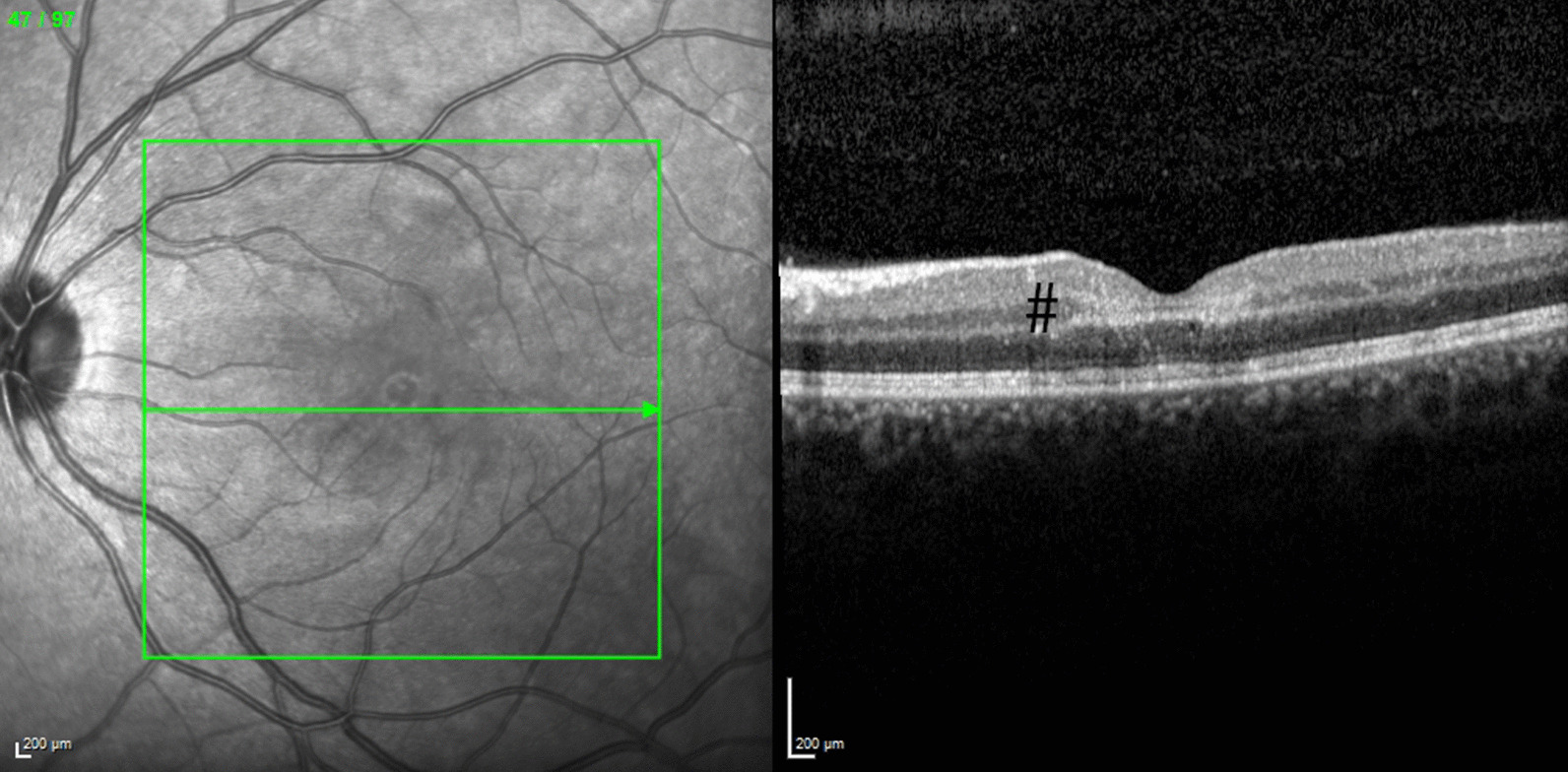
Heidelberg SD-OCT of the paramacular lesion revealing a hyperreflectivity at the junction of the outer plexiform layer (OPL) and inner nuclear layer (INL), black # revealing a focal ischemia

The patient was admitted and required urinary catheterization. She was urgently referred to the ophthalmology department, and fundoscopy showed bilateral parafoveolar microhemorrhages, and whitish lesions of the retina (Fig. 2A). Visual acuity was 0.3 (right eye) and 0.4 (left eye). Optical coherence tomography angiography revealed superficial and deep microangiopathy of the retina (Fig. 2B–D). Heidelberg spectral domain optical coherence tomography (SD-OCT) of the paramacular lesion revealed a hyperreflectivity at the junction of the outer plexiform layer and inner nuclear layer, revealing a focal ischemia (Fig. 3). The diagnosis of Behçet’s disease was made, and intravenous corticosteroid treatment was initiated with pulsed 500 mg/day methylprednisolone infusion for 3 days, followed by systemic oral prednisone 1 mg/kg/day with a tapering schedule. Progressive improvement of visual acuity and healing of vulvar ulcers were observed over the days following treatment initiation. Her estroprogestative pill was switched to a progestative-only pill owing to the risk of thrombosis associated with Behçet’s disease. The patient was discharged from hospital on day 4. Within 48 hours, a TNF blocking agent (intravenous infliximab 5 mg/kg/dose) was started. Visual acuity improved progressively. Infliximab was administered for a total of 4 months along with oral azathioprine up to 2 mg/kg/day. She will likely continue azathioprine for long-term maintenance, once corticosteroid tapering is completed. At the last visit, one and a half years after the initial symptoms, the patient had not experienced any relapse. At the last visit, Heidelberg SD-OCT (Fig. 3 right) showed atrophy of the macula. Besides, OCTA images remained unchanged, but the patient had recovered a vision of 1.0 (20/20) in both eyes.

## Discussion

### Behçet’s disease

Behçet’s disease is a systemic vasculitis that involves blood vessels of any size, affecting young adults at a mean age of 34 years. Its incidence is 0.38 per 100,000 in North America and Western countries [1, 3]. This disease is associated with immune dysfunction, mainly within the innate immune system, resulting in peculiar neutrophilic activation, abnormal endothelial activation, altered T-cell response, and cytokine release. HLA-B51 is more often, but not always, expressed among patients affected with Behçet’s syndrome [4]. It is more frequent in the Mediterranean regions and Asia (ancient Silk Road). As shown in the present case, however, Behçet’s disease can also occur in Western-European patients, suggesting significant environmental factors, such as infectious triggers [5].

### Clinical manifestations

Clinical manifestations include oral aphthosis (95% of the patients), recurrent genital ulceration (60–90%), pseudofolliculitis or erythema nodosum (0–90%), uveitis or retinal vasculitis (45–90%), gastrointestinal ulceration (4–38%), venous and arterial thrombosis or aneurysm (2.2–50%), and neurological (2.5–38.5%) and articular symptoms (11.6–93%). A positive pathergy test, consisting in a pustule forming on the skin at the site of a needle puncture, can also be observed and, if present, is a highly specific sign of Behçet’s disease. The diagnosis of Behçet’s disease is based on clinical criteria, some of which are assembled as a score [6]. The diagnostic criteria are summarized in Table 2. In our patient, a score of 2 points for genital ulcers and 2 points for ocular lesions supported a diagnosis of Behçet’s disease. There is no reliable laboratory marker to support the diagnosis of Behçet’s disease, although the presence of HLA-B51 may be suggestive [6].

**Table 2:** International Criteria for Behçet’s Disease—point score system: scoring > 4 indicates Behçet’s disease(6)

Clinical manifestation	Point(s)
Ocular lesions	2
Genital aphthosis	2
Oral aphthosis	2
Skin lesions	1
Neurological manifestations	1
Vascular manifestations	1
Positive pathergy test	1

### Genital ulcers

After oral aphthosis, genital ulceration is the most frequent symptom of Behçet’s disease. Genital ulcers in Behçet’s disease are generally multiple, confluent, and well defined with a surrounding red halo. Yellowish pseudomembranes are often observed. They are typically recurrent and painful, and tend to scar [7]. Many other conditions can cause genital ulcers: infectious (HSV, syphilis, chancroid, granuloma inguinale, lymphogranuloma venereum, HIV), noninfectious, bullous, traumatic, neoplastic, and drug-related causes [8]. Clinically, PCR samples and serologies can help to rule out an infection. Histology of Behçet’s ulcer is generally of little or no help, being very variable and nonspecific [7], and therefore the diagnosis should be made clinically. As presented in our case, complications such as urinary retention can appear, similarly to primary HSV infection.

Our patient consulted the gynecological emergency service twice as her genital ulcers were her most concerning and painful symptom. In the presence of genital ulcers associated with vision impairment, the gynecologist should consider the diagnosis of inaugural Behçet’s disease and call an ophthalmologist urgently, due to the possible rapid evolution towards complete blindness.

### Ocular symptoms

Ocular symptoms of Behçet’s disease may be associated with a severe decrease of visual acuity or vision loss in up to 20% of the patients (due to macular lesions, retinal-occlusive disease, or severe panuveitis) in the absence of prompt treatment [2]. Typically, patients present with panuveitis and hypopyon.

Different tools allow the for diagnosis of ocular lesions. Fundoscopy (Fig 2A) allows general exploration of the retina. In our case, it showed retinal vasculitis and parafoveolar microhemorrhages, related to macular vaso-occlusive disease (macular and nasal lesion). These typical lesions are highly correlated with Behçet’s disease according to uveitis expert specialists [9]. Optical coherence tomography angiography (OCTA) (Fig 2B–D) may provide images of retinal and choroidal vasculature based on temporal signal change due to erythrocyte movement in the vessels. It assesses for perifoveolar microvascular changes, alterations of the superficial and deep vascular plexuses of the retina recently observed in Behçet’s disease. In our case, OCTA revealed vaso-occlusive lesions of the deep and superficial retinal capillary plexuses, which is highly suggestive of ocular Behçet’s disease [10].

In Behçet’s disease, which is an ophthalmological emergency, prompt therapy with intravenous methylprednisolone is necessary to reverse the inflammatory process and avoid further retinal damage.

### Thrombotic risk

Vascular symptoms, if present, involve vessels from capillaries to large vessels. There is a risk of phlebitis, as well as deep vein thrombosis [11]. Given the additional risk of vascular events caused by Behçet’s disease [12], although there are no clear guidelines about contraception in patients with Behçet’s disease, it seems reasonable to prefer progestative-only methods in these patients with additional vascular risk [13].

### Treatment

The management of this inaugural Behçet’s disease required a rapid initiation of high doses of intravenous corticosteroids and anti-TNF therapy. Intravenous or oral corticosteroids are the treatment of choice for acute systemic manifestations of Behçet’s disease. Their dose and duration or association with other molecules depend on the clinical manifestations of the disease and its severity [14]. Recently, immunomodulators have gained importance in the treatment of Behçet’s disease [15]. Colchicine classically constitutes the first-line treatment for the long-term prevention of mucocutaneous lesion recurrences, but is not effective enough to treat eye involvement [16]. Apremilast, an inhibitor of phosphodiesterase-4, showed efficacy in decreasing mucocutaneous ulcers. Infliximab, an anti-TNF-alpha agent, may be considered as second-line treatment for severe ocular Behçet’s disease after treatment of the acute phase by intravenous methylprednisolone [15].

## Conclusion

The differential diagnosis of genital ulcers is broad and, thus, a major clinical challenge for the gynecologist. Behçet’s disease, although rare in Western countries, needs to be suspected when genital ulcers are associated with visual symptoms. Ophthalmologic management is an emergency in Behçet’s disease since retinal microangiopathy, even if uncommon, can lead to irreversible vision impairment in young adults.

## Data Availability

All relevant data are contained in the article. There are no supplementary data.

## Abbreviations

CRP: C-reactive protein
EBV: Epstein–Barr virus
HIV: Human immunodeficiency virus
HLA: Human leukocyte antigen
HSV: Herpes simplex virus
IV: Intravenous
PCR: Polymerase chain reaction
TNF: Tumor necrosis factor
